# Dysregulation of intestinal epithelial electrolyte transport in canine chronic inflammatory enteropathy and the role of the renin-angiotensin-aldosterone-system

**DOI:** 10.3389/fvets.2023.1217839

**Published:** 2023-08-31

**Authors:** Franziska Dengler, Oliver Domenig, Stefanie Kather, Iwan A. Burgener, Joerg M. Steiner, Romy M. Heilmann

**Affiliations:** ^1^Institute of Physiology, Pathophysiology and Biophysics, University of Veterinary Medicine Vienna, Vienna, Austria; ^2^Attoquant Diagnostics, Vienna, Austria; ^3^Department for Small Animals, College of Veterinary Medicine, University of Leipzig, Leipzig, SN, Germany; ^4^Small Animal Internal Medicine, University of Veterinary Medicine Vienna, Vienna, Austria; ^5^Gastrointestinal Laboratory, School of Veterinary Medicine and Biomedical Sciences, Texas A&M University, College Station, TX, United States

**Keywords:** diarrhea, dog, inflammatory bowel disease, intestinal epithelial transport, mRNA expression, mass spectrometry, RAAS, SGLT1

## Abstract

Chronic diarrhea is a hallmark sign of canine chronic inflammatory enteropathy (CIE), leading to fluid and electrolyte losses. Electrolyte homeostasis is regulated by the renin-angiotensin-aldosterone-system (RAAS), which might be involved in (counter-)regulating electrolyte losses in canine CIE. Whether and which electrolyte transporters are affected or if RAAS is activated in canine CIE is unknown. Thus, intestinal electrolyte transporters and components of the RAAS were investigated in dogs with CIE. Serum RAAS fingerprint analysis by mass spectrometry was performed in 5 CIE dogs and 5 healthy controls, and mRNA levels of intestinal electrolyte transporters and local RAAS pathway components were quantified by RT-qPCR in tissue biopsies from the ileum (7 CIE, 10 controls) and colon (6 CIE, 12 controls). Concentrations of RAAS components and mRNA expression of electrolyte transporters were compared between both groups of dogs and were tested for associations among each other. In dogs with CIE, associations with clinical variables were also tested. Components of traditional and alternative RAAS pathways were higher in dogs with CIE than in healthy controls, with statistical significance for Ang I, Ang II, and Ang 1–7 (all *p* < 0.05). Expression of ileal, but not colonic electrolyte transporters, such as Na^+^/K^+^-ATPase, Na^+^/H^+^-exchanger 3, Cl^−^ channel 2, down-regulated in adenoma, and Na^+^-glucose-cotransporter (all *p* < 0.05) was increased in CIE. Our results suggest that the dys- or counter-regulation of intestinal electrolyte transporters in canine CIE might be associated with a local influence of RAAS. Activating colonic absorptive reserve capacities may be a promising therapeutic target in canine CIE.

## 1. Introduction

Both inflammatory bowel disease (IBD) in humans and chronic inflammatory enteropathy (CIE) in dogs show an increasing incidence that poses an immense burden on the healthcare system (1). A frequent clinical sign that significantly negatively impacts the patient’s quality of life is chronic diarrhea, the discharge of loose to watery feces causing electrolyte losses, plasma electrolyte shifts, and secondary systemic consequences (2, 3). Because the intestinal absorption of electrolytes is extensively regulated, it would be expected that counterregulatory mechanisms to balance fluid and electrolyte deficiencies caused by the disease are activated. A major regulator of electrolyte and fluid homeostasis is the renin-angiotensin-aldosterone system (RAAS), which controls systemic blood pressure and volume by regulating vascular tone and the reabsorption of Na^+^ and water. Beyond this traditional systemic role, RAAS has recently gained increasing attention as a local autocrine and paracrine mediator. Within that scope, RAAS effects beyond the classical modulation of (primarily renal and intestinal) electrolyte transport may include developing and perpetuating inflammation and fibrotic tissue remodeling (4, 5). An upregulation of individual components of RAAS has been observed in human IBD patients (6). Altered expression patterns and dysfunction of several ion transporters, especially Na^+^/H^+^-exchanger (NHE) 3, epithelial Na^+^-channel (ENaC), Na^+^/K^+^-ATPase, down-regulated in adenoma (DRA), and putative anion transporter 1 (PAT1), appear to occur in human patients with IBD (7–9). This might be a consequence of inflammatory signaling or be regulated directly or indirectly by the multimodal effects of RAAS and contribute to the plasma electrolyte shifts observed in IBD patients. While hyponatremia is the most important electrolyte shift in human IBD patients, dogs with CIE more commonly show hypokalemia (3), suggesting differences in the pathophysiology and compensatory mechanisms between species. This difference may also be associated with a difference in the primary localization of the inflammatory lesions along the gastrointestinal tract between human IBD and canine CIE patients. However, neither the possible activation of RAAS nor lack thereof or the intestinal expression of electrolyte transporters has been investigated in canine CIE thus far.

As a prelude to further functional analyses, our study aimed to investigate (i) the gene expression of intestinal electrolyte transporters with a putative role in salvaging or contributing to electrolyte losses in dogs with CIE, (ii) the possibility of activation of the RAAS in dogs with CIE, and (iii) the possible association between the expression of intestinal electrolyte transporters and currently known RAAS components.

## 2. Materials and methods

### 2.1. Animals

Healthy control dogs were required to be free from having any clinical signs of gastrointestinal disease or receiving medication known to affect the gastrointestinal tract, to be regularly vaccinated and dewormed, and – for the serum controls – to have a normal complete blood cell count and serum biochemistry profile.

Dogs with CIE had clinical signs of chronic enteropathy (i.e., vomiting, diarrhea, and/or weight loss for ≥3 weeks); other possible etiologies of these clinical signs (e.g., atypical hypoadrenocorticism, exocrine pancreatic insufficiency) were excluded. Intestinal inflammation was documented histologically, and the response to treatment supported a diagnosis of CIE (10). These dogs could not have (i) received any anti-inflammatory and/or immunosuppressive medication or RAAS-acting agent (i.e., angiotensin-converting enzyme [ACE] inhibitor, angiotensin receptor blocker, aldosterone-antagonist or other diuretic, ß-blocker, calcium channel blocker, or mineralocorticoid) within 4 weeks prior to enrollment and sampling and (ii) evidence of cardiac disease or renal insufficiency. Clinical disease severity was determined using the canine chronic enteropathy clinical activity index (CCECAI), which includes the 3-point-scale evaluation of the dog’s attitude/activity, appetite, frequency of vomiting, stool consistency, frequency of defecation, weight loss, serum albumin concentration, peripheral edema/ ascites, and pruritus score (11). Gastrointestinal tissue biopsies were histologically evaluated by a 3-point scale grading structural and inflammatory lesions (12).

Serum samples (*n* = 5) and endoscopic tissue biopsies from the ileum (*n* = 7) and colon (*n* = 6) of dogs with CIE were from a previous study (13) that was approved by the Regional Council of the State of Saxony, Chemnitz/Leipzig, Germany (#TVV 06–17). Control tissues were full-thickness biopsies from the ileum (*n* = 10) and colon (*n* = 12) of purpose-bred healthy dogs that were euthanized for an unrelated project at the School of Veterinary Medicine and Biomedical Sciences at Texas A&M University, United States (Animal Use Protocol #TAMU 2009–0123). Serum from age- and sex-matched healthy controls (*n* = 5) were surplus materials from the blood donor bank at the Department for Small Animals, University of Leipzig, Germany.

### 2.2. Real-time quantitative polymerase chain reaction (RT-qPCR) analyses

Biopsies were stored in RNAlater® (Qiagen, Hilden, Germany) at −80°C until RNA isolation. Total RNA was isolated from the tissue, and cDNA was prepared as described previously (14). Briefly, total RNA was extracted with the ReliaPrep™ RNA Miniprep System (Promega GmbH, Mannheim, Germany) according to the manufacturer’s protocol, including treatment with DNase. RNA concentration and quality were determined spectrophotometrically (DeNovix DS-11, Wilmington, DE, United States), and 1 μg of high-quality RNA was used for cDNA synthesis using the GoScript™ Reverse Transcriptase Kit (Promega, Mannheim, Germany).

For qPCR, a ready-to-use SYBR green master mix (GoTaq^®^, Promega, Mannheim, Germany) with 112 nM primer mix was used. Primers were designed with the Primer BLAST tool from the National Center for Biotechnology Information (NCBI, Bethesda, MD, United States) using known sequences from the Basic Local Alignment Search Tool (BLAST) in the NCBI gene bank database (Table 1) and were synthesized by Microsynth (Balgach, SG, Switzerland). A no-template control (NTC) with DNase-free water instead of cDNA was included in each run. For each sample and gene, qPCR reactions were run in duplicates, and the amplification specificity was checked by melting curve analysis. Following denaturation at 95°C, extension and annealing were performed at 60°C, and the quantification cycle was determined using the CFX Maestro software (Biorad, Vienna, Austria). The ΔΔC_t_ method was used to analyze the data and compare the mRNA expression of the electrolyte transporters Na^+^/K^+^-ATPase (*ATP1A1*), *ENAC*, *NHE3*, renal outer medullary K^+^ channel (*ROMK*), cystic fibrosis transmembrane conductance regulator (*CFTR*), *NHE2*, *CLC-2*, *DRA*, monocarboxylate transporter 1 (*MCT1*), and Na^+^-glucose co-transporter 1 (*SGLT1*); as well as the local RAAS components angiotensin II receptor type 1 (*AGTR1*) and a disintegrin and metalloproteinase (*ADAM*). Samples were normalized using the same amounts of RNA and cDNA for processing. Hypoxanthine guanine phosphoribosyltransferase 1 (*HPRT1*), peptidylprolyl-isomerase D (*PPID*), and succinate dehydrogenase subunit A (*SDHA*) served as reference genes after their stability was confirmed using Reffinder (15). The geometric mean of all reference genes’ C_t_ values was calculated for each sample and used for normalization. Additionally, vimentin (*VIM*) expression was used to normalize mesenchymal tissue content across intestinal biopsy specimens.

**Table 1:** Primers used for qPCR.

Gene name	Gene bank accession no.	Primer sequence (5′ – 3′)
*ATP1A1*	NM_001389224.1	F: ACTCAGAACCGGATGACCGT R: ATCGAACGAGACACCACTCTG
*AE2*	XM_003639553.5	F: AGAGCAAGCGGGTTATGCC R: AGAAAGAATCTGCGCCCGAG
*CFTR*	NM_001007143.1	F: GGACAGAGAGCTGGCATCAA R: CTGCTTTGGTGACTTCCCCT
*CLC2*	XM_038445731.1	F: CCGGTCTTTGTTATCGGAGC R: ATCCGGTAAGTGCTGCTGTC
*DRA*	XM_038423776.1	F: CTGGGATTCTCTCTGCGGTC R: GAGCTGCCAGGACAGACTTTT
*ENAC*	XM_038439216.1	F: GGGATCAAAAATGGCCTGTCC R: CATCCTGCCCATGCACCATT
*HPRT1*	NM_001003357.2	F: CCCAGCGTCGTGATTAGTGA R: CACTTTTTCCAAATCCTCAGCGT
*MCT1*	XM_038423242.1	F: CCGCGCATAACGGTATTTGG R: CCTCCATCTGGGGGAGTGTA
*MCT4*	XM_038675356.1	F: ATCGTGGGCACCCAGAAGTT R: CAAGAGCTTGCCTCCCGAT
*NHE2*	XM_038680184.1	F: CCCTGGCGAAGATAGGCTT R: CTAGCAGCAGGCCAACCATT
*NHE3*	XM_038462883.1	F: GTGGTCACCTTCAAATGGCAC R: GTGTGATAGGTGGAAGCCGAT
*PPID*	XM_038688725.1	F: TGGAAATGTCGCATCCGTCC R: CAATTCGACCAACTCGCTCC
*ROMK*	XM_038664232.1	F: GCACGCACTCTCCAGATCAGA R: CTTTGCCGAGAATGCCCAAA
*SDHA*	XM_535807.6	F: TCCGTGTGGGAAGTGTGTTA R: GTGTTCCAGACCATTCCTCG
*VIM*	NM_001287023.1	F: GGATGCACTCAAAGGGACTAATG R: GTCTTGGTAGTTAGCAGCTTCG

### 2.3. Mass spectrometry-based serum RAAS profiling

Serum samples stored at −20°C were submitted to a commercial laboratory (Attoquant Diagnostics, Vienna, Austria) to quantify equilibrium concentrations of RAAS components (angiotensin I [Ang I], Ang II, Ang III, Ang IV, Ang (1-7), Ang (1-5), and aldosterone) by liquid chromatography–tandem mass spectrometry (LC–MS/MS) as previously described (16, 17). Briefly, serum samples were equilibrated for 1 h at 37°C prior to stabilization, spiked with stable isotope-labeled Ang peptides and deuterated aldosterone (internal standard), subjected to C18-based solid-phase extraction and LC–MS/MS analysis using a reverse-analytical column in a triple quadrupole MS (Xevo TQ-S; Waters, Eschborn, Germany). After normalizing the recovered RAAS components against their internal standards, the concentrations of these analytes were calculated based on calibration curves using integrated chromatograms (16, 17). PRA-S (a marker for serum renin activity), ACE-S (a marker for serum ACE activity), AA2 ratio (a marker for adrenocortical responsiveness), and ALT-S (a marker for renin-independent alternative serum RAAS activity) were calculated as described (18–20).

### 2.4. Statistics

A commercial statistical software program (JMP® v.13, SAS Institute, Cary, NC, United States) was used for all statistical analyses. Data were assessed for normality using a Shapiro–Wilk test, and the summary statistics were reported as medians and ranges (continuous data) or counts and percentages (categorical data). Non-parametric two-group comparisons were performed using a Wilcoxon rank-sum test, and a Spearman correlation coefficient (ρ) was calculated to assess for possible correlations. Statistical significance was set at *p* < 0.05 and a Bonferroni correction for multiple comparisons was applied if indicated.

## 3. Results

### 3.1. mRNA expression of electrolyte transporters and local RAAS components

**Figure 1:**
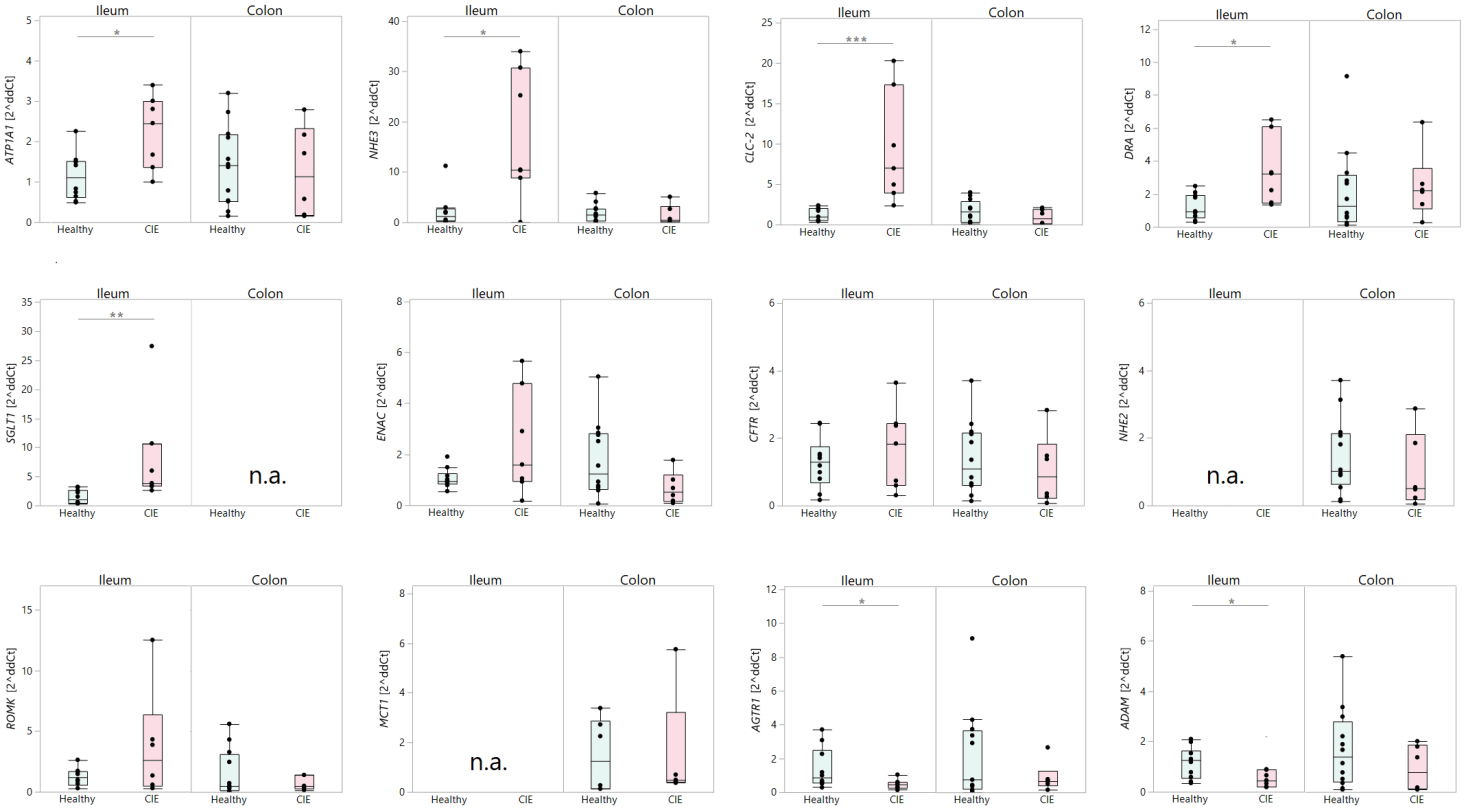
Electrolyte transporter and selected tissue RAAS component mRNA expression in the ileal and colonic mucosa from dogs with CIE compared to healthy controls. The expression of ileal *ATP1A1, NHE3*, *CLC-2, DRA*, and *SGLT1* mRNA was increased in dogs with CIE (all *p* < 0.05), whereas there were no differences in the expression of *ENAC, CFTR*, and *ROMK* mRNA in the ileum. No differences in the expression of any of the transporters were detected in the colonic mucosa. Local RAAS imbalance comprised a downregulated ileal (but not colonic) *AGTR1* and *ADAM* mRNA expression in dogs with CIE (^*^ indicates significance at *p* < 0.05, ^**^ indicates significance at *p* < 0.01, ^***^ indicates significance at *p* < 0.001). *ADAM*: a disintegrin and metalloproteinase; *AGTR1*: angiotensin II receptor type 1; *ATP1A1*: Na^+^/K^+^-ATPase; *CFTR*: cystic fibrosis transmembrane conductance regulator; *CLC-2*: Cl^−^ channel 2; *DRA*: down-regulated in adenoma; *ENAC*: epithelial Na^+^ channel; *MCT1*: monocarboxylate transporter 1; *NHE2*: Na^+^/H^+^-exchanger 2; *NHE3*: Na^+^/H^+^-exchanger 3; *ROMK*: renal outer medullary K^+^ channel; *SGLT1*: Na^+^-glucose co-transporter 1; n.a.: not abundant.

Significant upregulation of the ileal epithelial mRNA expression of the electrolyte transporters *ATP1A1* (*p* = 0.0218), *NHE3* (*p* = 0.0491), *CLC-2* (*p* = 0.0009), and *DRA* (*p* = 0.0128), along with the glucose transporter *SGLT1* (*p* = 0.0015), was detected in dogs with CIE compared to healthy controls (Figure 1A). No significant difference was observed for ileal *ENAC* (*p* = 0.1571), *ROMK* (*p* = 0.3562), and *CFTR* (*p* = 0.6961) expression between CIE and healthy controls. Furthermore, no differences were detected between both groups of dogs in the colonic epithelial expression of *ATP1A1* (*p* = 0.8513), *NHE3* (*p* = 0.4260), *ROMK* (*p* = 1.0000), *CFTR* (*p* = 0.4824), *DRA* (*p* = 0.6734), *MCT1* (*p* = 0.6473), *ENAC* (*p* = 0.2059), *CLC-2* (*p* = 0.2229), and *NHE2* (*p* = 0.3024) in dogs with CIE compared to the control group (Figure 1A).

Significant downregulation of the ileal epithelial *AGTR1* and *ADAM* expression (both *p* = 0.0359) was seen in dogs with CIE compared to healthy controls (Figure 1B), which was also not mirrored in the colon (*p* = 0.7078 and *p* = 0.3487).

### 3.2. Serum concentrations of RAAS components

**Figure 2:**
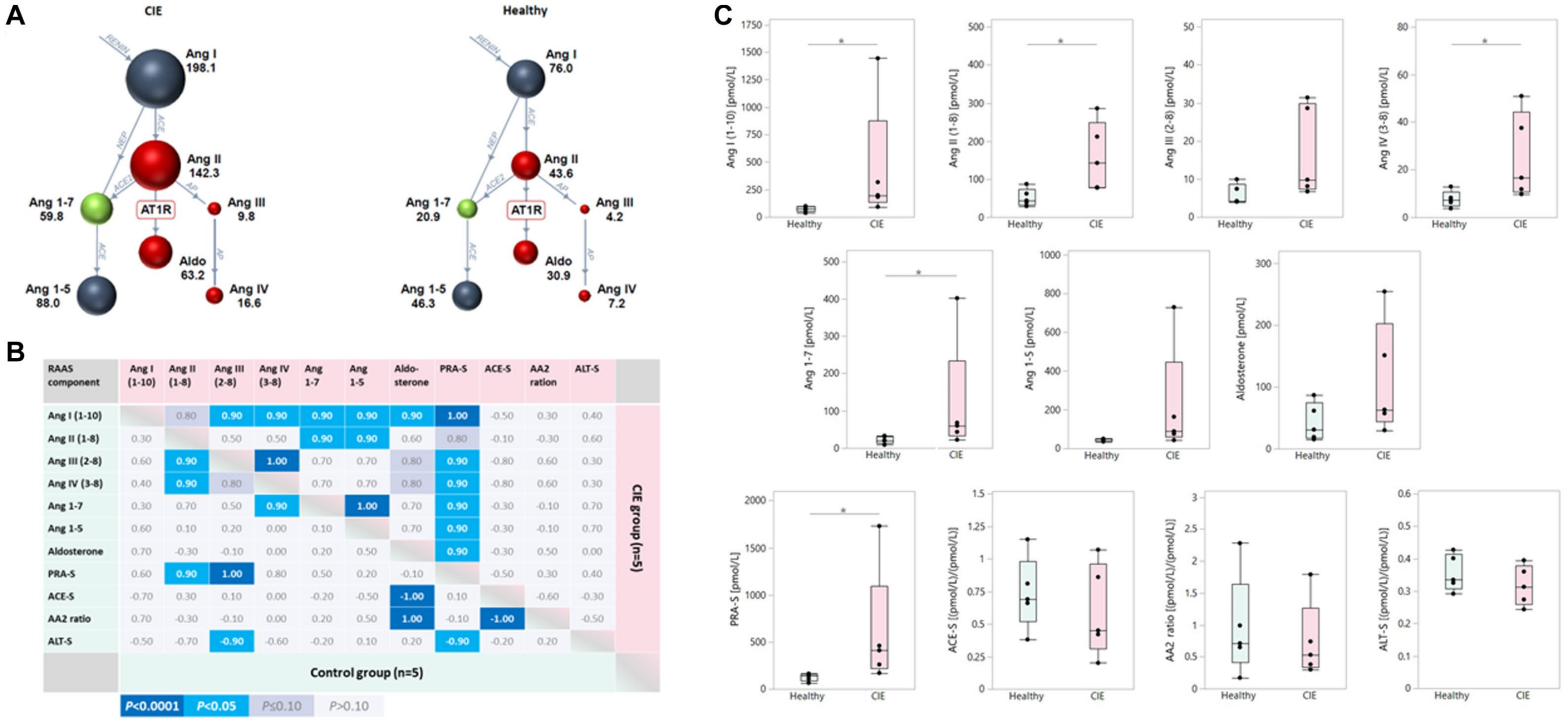
Upregulation of serum RAAS components in canine CIE. **(A)** Serum RAAS fingerprint visualizing the upregulation of both the traditional and alternative RAAS arms in dogs with CIE (*n* = 5; left panel) compared to healthy controls (*n* = 5; right panel). Individual colored dots (and numbers) reflect the median concentrations for each analyte. **(B)** Correlation among serum RAAS components in dogs with CIE and healthy controls. The Spearman correlation coefficients (ρ) for the individual correlations are shown, with the color scheme (indicated below the table) reflecting the respective significance levels for each correlation. **(C)** Two-group comparisons for the individual analytes in serum show traditional and alternative RAAS components to be increased in dogs with CIE compared to controls, with statistical significance reached for Ang I, Ang II, Ang IV, and Ang 1–7 as well as PRA-S (* indicates significance at *p* < 0.05). Ang I (1-10): angiotensin I; Ang II (1-8): angiotensin II; Ang III (2-8): angiotensin III; Ang IV (3-8): angiotensin IV; Ang 1–7: angiotensin 1–7; Ang 1–5: angiotensin 1–5; PRA-S: marker of plasma renin activity (calculated); ACE-S: ratio of Ang II (1-8) to Ang I (1-10) = marker of angiotensin-converting enzyme (ACE) activity; AA2 ratio: ratio of aldosterone to Ang II (1-8) = marker of adrenal responsiveness to Ang II (1-8); ALT-S: ratio of [Ang 1–7 + Ang 1–5] to [Ang I (1-10) + Ang II (1-8) + Ang 1–7 + Ang 1–5] = marker of renin-independent alternative serum RAAS activity.

Components of both traditional and alternative RAAS pathways were increased in serum from dogs with CIE compared to healthy control dogs (Figure 2A). Ang I, Ang II, Ang IV, and Ang 1–7 as well as PRA-S were significantly upregulated in CIE (all *p* = 0.0367) compared to healthy dogs, whereas statistical significance was not reached for the differences in serum Ang III and Ang 1–5 (both *p* = 0.0947) or aldosterone (*p* = 0.2101; Figure 2B). Mildly but insignificantly decreased ACE-S and AA2 ratios (both *p* = 0.6761) and ALT-S (*p* = 0.2963) were seen in dogs with CIE. Traditional and alternative RAAS components were highly correlated in dogs with CIE, with few significant correlations in healthy dogs (Figure 2C).

### 3.3. Association of serum RAAS components with patient characteristics and intestinal electrolyte transporter and local RAAS component mRNA expression

**Figure 3:**
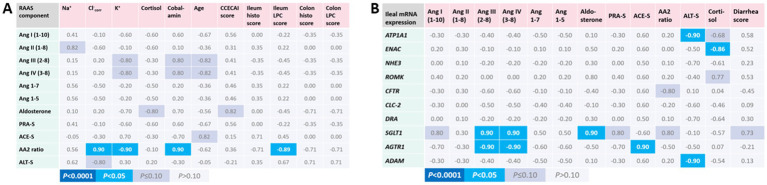
Association of serum RAAS activation with patient and disease characteristics, intestinal electrolyte transporter, and local RAAS component expression in canine CIE. **(A)** Correlations of serum RAAS components with patient and disease characteristics. Only the calculated serum AA2 ratio was significantly positively correlated with serum corrected Cl^−^ and cobalamin concentrations and was inversely correlated with serum K^+^ concentrations and histologic lesion scores in the ileum. **(B)** Correlations of serum RAAS components with the mRNA expression of electrolyte transporters, *ADAM*, and *AGTR1*. Shown are the Spearman correlation coefficients (ρ) for the individual correlations, with the color scheme (indicated below the table) reflecting the respective significance levels for each correlation.

Few correlations of serum RAAS components with patient and disease characteristics were seen in the CIE group of dogs (Figure 3A). Lower serum AA2 ratios, reflecting decreased adrenal responsiveness to Ang II, were strongly correlated with higher K^+^, lower corrected Cl^−^, higher histologic lesion scores in the ileum, and lower serum cobalamin concentrations. Notably, *SGLT1* mRNA expression was highly correlated with several RAAS components (Ang I, Ang III, Ang IV, aldosterone, PRA-S, and AA2 ratio) and the severity of diarrhea in dogs with CIE (Figure 3B). No other significant correlations were seen between the expression of ileal electrolyte transporters, endogenous glucocorticoid (serum cortisol), and RAAS components in the serum.

## 4. Discussion

Chronic diarrhea is a hallmark of canine CIE and a major factor impairing affected dogs’ and their owners’ quality of life. Thus, a more detailed understanding of the mechanisms involved in the pathogenesis of diarrhea and the corresponding counterregulatory mechanisms is of urgent interest to improve the therapeutic approach in affected dogs. This preliminary study aimed to characterize possible changes in the expression of intestinal electrolyte transporters in canine CIE and to investigate the potential role of RAAS in their regulation.

The results demonstrate an upregulation of electrolyte transporter mRNA expression in the ileum, which is usually more affected than the colon in canine CIE. Particularly the Na^+^-transporters *ATP1A1* and *NHE3* were significantly upregulated in dogs with CIE compared to healthy dogs. *ENAC* appeared numerically upregulated, but the difference between CIE and healthy controls did not reach statistical significance. This upregulation could be interpreted as a compensatory attempt to increase Na^+^ and thus also water absorption in the diseased intestine. *DRA* was also increased in CIE and might be involved in NaCl absorption, cooperating with *NHE3* (7), and contribute to water reabsorption. These findings contrast with the reduced expression and function of *DRA, ENAC*, and *ATP1A1* in rodent colitis models and human IBD patients (7–9, 21–25), suggesting species-specific differences in the dysregulation or counter-regulation of these transporters.

Another explanation for these discrepant results among dogs, humans, and experimental rodents might be the primary location of the disease. Human IBD, including Crohn’s disease (CD) and ulcerative colitis, mostly affects the colon and disease localization strictly to the ileum is seen in only approximately 30% of CD cases (26). In contrast, canine CIE has a more heterogenous distribution along the gastrointestinal tract and lesions often predominate in the small intestine, particularly in the ileum (27, 28). Along with these species-specific differences, it is reasonable to assume that the sites and mechanisms of dysregulation or counter-regulation would also differ. In human CD, the differentiation between ileal and colonic disease has been proposed to account for differences in the pathogenesis, optimal therapeutic approaches, and the corresponding treatment success rates (26, 29). Thus, the results obtained for canine CIE may generally resemble human ileal CD more closely than CD with predominantly colonic inflammatory lesions. Differential investigation of the transepithelial electrolyte transport in human ileal and colonic CD is lacking thus far and these hypotheses warrant further study in canine CIE and human IBD.

Upregulation of the NaCl uptake mechanisms might be an attempt to compensate for electrolyte and free water losses associated with the maldigestion and malabsorption resulting from the exacerbated immune response of the intestinal mucosa. These counterregulatory mechanisms could be induced, exacerbated, or otherwise modulated by the RAAS as a regulator of systemic blood pressure activated by low plasma NaCl concentrations (30). A regulatory effect of RAAS on colonic electrolyte transport has been established, specifically an upregulation of *ENAC* and *ATP1A1* by aldosterone (31). This regulatory effect appears to be mediated both on the functional and transcriptional level (32). Also, an upregulation of NHE3 in the proximal colon, but not the ileal or renal epithelium, was demonstrated in aldosterone-treated rats (33). These transport mechanisms stimulate NaCl and free water absorption, leading to an increase in blood pressure. Stimulation of the Na^+^ absorption by RAAS would also explain the predominance of hypokalemia in dogs with CIE (3), as K^+^ losses usually accompany renal and intestinal Na^+^ absorption and is in line with the strong inverse correlation of K^+^ plasma levels with serum AA2 ratios we found in this study. Human IBD, in contrast, is mostly associated with hyponatremia (3), further supporting our hypothesis of differential regulation of electrolyte transporters in canine CIE and human IBD.

In addition to aldosterone, Ang II has been shown to affect intestinal electrolyte absorption (34), such as via activation of ENaC by AGTR1 stimulation (34). However, its effects may be dose- and receptor-dependent (34, 35): while low to moderate Ang II levels stimulate Na^+^ and thus water absorption, presumably via the sympathetic and enteric nervous system, high levels of Ang II inhibit absorptive processes, which is proposed to be mediated by increased prostaglandin concentrations in the intestinal mucosa (35) and indicate an ambivalent effect of RAAS depending on the local mucosal microenvironment. As the primary site of intestinal RAAS-mediated regulation of electrolyte homeostasis, the colon has an enormous absorptive reserve capacity to compensate for fluid losses in the proximal gastrointestinal tract. Therefore, it is surprising that no changes in the expression of any colonic electrolyte transporters investigated – at least on the mRNA level – were seen in the dogs with CIE, given the finding of significant upregulation of the traditional and alternative RAAS arms in these dogs and their presentation with diarrhea of varying severity. We can only speculate as to the possible reasons for this finding. The extent of RAAS activation might not be sufficient to induce an upregulation of electrolyte transporter transcription in the colon, which would be consistent with the overall mild plasma electrolyte alterations reported (3). Furthermore, the sampling site of the colonic tissue biopsies might have been too far proximal, because the regulatory response was observed to vary between the proximal and distal colon in rats (36). In addition, serum concentrations of electrolytes and RAAS components might be affected by regulatory efforts of other organs (e.g., kidneys) and/or dietary Na^+^ concentrations.

Only very few significant correlations were detected between the overexpressed electrolyte transporter genes in the intestinal mucosa and the concentrations of RAAS components in serum. While this does not exclude a RAAS-mediated upregulation of the ileal electrolyte transporters, a direct effect on their transcription levels appears less likely. Still, translational or functional effects of certain traditional and/or alternative RAAS components could be mediated by AGTRs. In contrast, the strong correlation observed between mucosal *SGLT1* mRNA levels and several serum RAAS components supports a regulatory effect of RAAS on intestinal *SGLT1* expression. A relationship between SGLT1 activity and RAAS has been reported previously, but the effects of RAAS appear to be receptor-dependent (37–39). While activation of AGTR1 inhibits the activity of SGLT1, AGTR2 activation enhances SGLT1 activity (37). To our knowledge, an effect of RAAS on *SGLT1* gene expression has not previously been reported. Similar to the upregulation of *NHE3* and *ATP1A1*, an increased expression and activity of SGLT1 would enhance the absorption of osmotically active Na^+^ and glucose, thus ameliorating diarrhea. Whether this concept lends itself to an effective adjunct or even a sole nutritional approach requires further investigation.

The varying effects of RAAS on SGLT1 activity reported in the literature and the lack of correlations between the mucosal expression of most investigated electrolyte transporters and serum RAAS components in our study could also point to a local action of RAAS. Such a local RAAS activity could be mediated by the locally expressed AGTRs and be only partially dependent on or completely independent of systemic RAAS control. Local effects of the AGTR1-agonist losartan have been shown in the canine stomach (40), suggesting a functional local RAAS in the canine gastrointestinal tract. The downregulation of *AGTR1* in the inflamed ileal mucosa in canine CIE could be interpreted as a local counterregulatory mechanism to negate the increased availability of ligands either as a negative feedback loop or an effort to limit the effects of these ligands to specific target tissues. Compared to the results of this study on canine CIE, only some systemic RAAS components were found to be increased (ACE2, Ang 1–7, and Ang II), and aldosterone concentrations decreased in humans with IBD compared to healthy controls (6). Increasing evidence supports the existence of alternative (local) RAAS activity that counteracts the systemic effects of traditional RAAS pathways (41, 42). This alternative RAAS consists of small peptides derived from Ang I or II, such as Ang (1–9) and Ang (1–7), and additional receptors for RAAS components including Mas (4, 42, 43). While systemic RAAS effects are predominantly pro-inflammatory, these alternative pathways appear to be primarily anti-inflammatory and anti-fibrotic, promoting epithelial recovery (44). Whether these opposing effects apply to canine CIE and offer therapeutic potential, however, requires further investigation.

Similar to the ambivalent effects on local SGLT1 activity, binding of Ang II to AGTR1 elicits pro-inflammatory effects, whereas it may have anti-inflammatory effects via AGTR2 (45). Hence, differential regulation and pleiotropic effects of RAAS on intestinal mucosal electrolyte transport can be reasonably assumed.

In conclusion, our results indicate an upregulation of ileal, but not colonic, electrolyte transport mechanisms to enhance distal intestinal absorption of Na^+^ and free water in dogs with CIE. Hence, targeting colonic electrolyte uptake mechanisms to modulate its absorptive reserve capacity might pose a novel therapeutic avenue for CIE patients. We could also demonstrate an upregulation of traditional and alternative RAAS components in serum specimens from diarrheic dogs with CIE. Although a relationship between systemic RAAS and intestinal electrolyte transporter levels would appear plausible, a direct association and regulatory effects remain to be proven in future studies. Targeting the traditional and alternative RAAS pathways may be a novel therapeutic avenue to ameliorate diarrhea in dogs with CIE.

## Data availability statement

The original contributions presented in the study are included in the article/supplementary materials, further inquiries can be directed to the corresponding authors.

## Ethics statement

The animal study was reviewed and approved. Serum samples (*n* = 5) and endoscopic tissue biopsies from the ileum (*n* = 7) and colon (*n* = 6) of dogs with CIE were surplus materials from a previous study that was approved by the Regional Council of the State of Saxony, Chemnitz/Leipzig, Germany (#TVV 06–17). Control tissues were full-thickness biopsies from the ileum (*n* = 10) and colon (*n* = 12) of purpose-bred healthy dogs that were euthanized for an unrelated project at the School of Veterinary Medicine and Biomedical Sciences at Texas A&M University, United States (Animal Use Protocol #TAMU 2009–0123). Serum from age- and sex-matched healthy controls (*n* = 5) were surplus materials from the blood donor bank at the Department for Small Animals, University of Leipzig, Germany. Written informed consent was obtained from the owners for the participation of their animals in this study.

## Author contributions

FD and RMH: conception of the work and drafting the manuscript. FD, OD, SK, IAB, JMS, and RMH: acquisition, analysis, or interpretation of data, and revising the manuscript. All authors provide approval for publication of the content and agree to be accountable for all aspects of the work.

## Funding

This work was funded by a grant from the Leipzig veterinary junior scientist support program financed by the “Freundeskreis Tiermedizin,” the Faculty of Veterinary Medicine, and by Ceva Santé Animale.

## Conflict of interest

OD is employed by Attoquant Diagnostics.

The remaining authors declare that the research was conducted in the absence of any commercial or financial relationships that could be construed as a potential conflict of interest.

## Publisher’s note

All claims expressed in this article are solely those of the authors and do not necessarily represent those of their affiliated organizations, or those of the publisher, the editors and the reviewers. Any product that may be evaluated in this article, or claim that may be made by its manufacturer, is not guaranteed or endorsed by the publisher.
